# Artificial Intelligence Unveils the Unseen: Mapping Novel Lung Patterns in Bronchiectasis via Texture Analysis

**DOI:** 10.3390/diagnostics14242883

**Published:** 2024-12-21

**Authors:** Athira Nair, Rakesh Mohan, Mandya Venkateshmurthy Greeshma, Deepak Benny, Vikram Patil, SubbaRao V. Madhunapantula, Biligere Siddaiah Jayaraj, Sindaghatta Krishnarao Chaya, Suhail Azam Khan, Komarla Sundararaja Lokesh, Muhlisa Muhammaed Ali Laila, Vadde Vijayalakshmi, Sivasubramaniam Karunakaran, Shreya Sathish, Padukudru Anand Mahesh

**Affiliations:** 1 Department of Respiratory Medicine, JSS Medical College, JSS Academy of Higher Education & Research (JSS AHER), Mysore 570004, Karnataka, India; athiranair94@gmail.com (A.N.); drjayarajbs@yahoo.com (B.S.J.); chaya.sindghatta@gmail.com (S.K.C.); khan2201@gmail.com (S.A.K.); lokeshpulmo@gmail.com (K.S.L.); muhlisamr@gmail.com (M.M.A.L.); vijjichowdary6@gmail.com (V.V.); sivasubramaniam1810@gmail.com (S.K.); 2 Department of Community Medicine, JSS Medical College, JSS Academy of Higher Education & Research (JSS AHER), Mysore 570004, Karnataka, India; rakeshm468@gmail.com; 3 Center of Excellence in Molecular Biology and Regenerative Medicine (CEMR) Laboratory (DST-FIST Supported Center and ICMR Collaborating Center of Excellence—ICMR-CCoE), Department of Biochemistry (DST-FIST Supported Department), JSS Medical College, JSS Academy of Higher Education & Research (JSS AHER), Mysuru 570015, Karnataka, India; greeshmamv@jssuni.edu.in (M.V.G.); madhunapantulas@yahoo.com (S.V.M.); 4 Department of Radiology, JSS Medical College, JSS Academy of Higher Education & Research (JSS AHER), Mysore 570004, Karnataka, India; drdeepakbenny@gmail.com (D.B.); vikrampatil@jssuni.edu.in (V.P.); 5 Father Muller Medical College, Mangaluru 575002, Karnataka, India; shreyarathna26@gmail.com

**Keywords:** bronchiectasis, IMBIO, lung texture analysis, alveolar

## Abstract

**Background and Objectives:** Thin-section CT (TSCT) is currently the most sensitive imaging modality for detecting bronchiectasis. However, conventional TSCT or HRCT may overlook subtle lung involvement such as alveolar and interstitial changes. Artificial Intelligence (AI)-based analysis offers the potential to identify novel information on lung parenchymal involvement that is not easily detectable with traditional imaging techniques. This study aimed to assess lung involvement in patients with bronchiectasis using the Bronchiectasis Radiologically Indexed CT Score (BRICS) and AI-based quantitative lung texture analysis software (IMBIO, Version 2.2.0). **Methods:** A cross-sectional study was conducted on 45 subjects diagnosed with bronchiectasis. The BRICS severity score was used to classify the severity of bronchiectasis into four categories: Mild, Moderate, Severe, and tractional bronchiectasis. Lung texture mapping using the IMBIO AI software tool was performed to identify abnormal lung textures, specifically focusing on detecting alveolar and interstitial involvement. **Results:** Based on the Bronchiectasis Radiologically Indexed CT Score (BRICS), the severity of bronchiectasis was classified as Mild in 4 (8.9%) participants, Moderate in 14 (31.1%), Severe in 11 (24.4%), and tractional in 16 (35.6%). AI-based lung texture analysis using IMBIO identified significant alveolar and interstitial abnormalities, offering insights beyond conventional HRCT findings. This study revealed trends in lung hyperlucency, ground-glass opacity, reticular changes, and honeycombing across severity levels, with advanced disease stages showing more pronounced structural and vascular alterations. Elevated pulmonary vascular volume (PVV) was noted in cases with higher BRICSs, suggesting increased vascular remodeling in severe and tractional types. **Conclusions:** AI-based lung texture analysis provides valuable insights into lung parenchymal involvement in bronchiectasis that may not be detectable through conventional HRCT. Identifying significant alveolar and interstitial abnormalities underscores the potential impact of AI on improving the understanding of disease pathology and disease progression, and guiding future therapeutic strategies.

## 1. Introduction

Bronchiectasis is a chronic pulmonary disorder characterized by the permanent dilation and destruction of the bronchial walls, leading to impaired mucociliary clearance and recurrent infections [1]. Despite advancements in respiratory medicine, bronchiectasis remains underdiagnosed and poses significant challenges to patient management, severely affecting quality of life. Once considered rare, recent epidemiological data indicate a rising prevalence, especially among older adults and individuals with comorbid respiratory conditions such as chronic obstructive pulmonary disease [COPD] and cystic fibrosis. In Europe and North America, the prevalence of bronchiectasis has been reported to range from 67 to 566.1 per 100,000 inhabitants [2,3,4,5], and, in China, among those aged 40 years and older, it reaches as high as 1200 per 100,000 [6].

The European Multicenter Bronchiectasis Audit and Research Collaboration [EMBARC] India registry highlights that males are more frequently affected than females, with a median patient age of 56 years [IQR 41–66]. Compared to Western registries, pulmonary tuberculosis [PTB] remains a leading cause of bronchiectasis in India, while allergic bronchopulmonary aspergillosis [ABPA] contributes significantly [8.9%] to disease etiology. Pseudomonas aeruginosa is commonly isolated in sputum cultures, affecting 13.7% of cases [7].

The pathogenesis of bronchiectasis is multifactorial, involving a complex interplay between infections, immune responses, and airway structural abnormalities. Etiologies include post-infectious complications, genetic disorders such as cystic fibrosis, immunodeficiency, and environmental exposures [8]. Despite the advent of high-resolution computed tomography [HRCT], which has revolutionized the detection and characterization of bronchiectasis, a disconnect persists between HRCT findings and clinical parameters, such as symptom severity, exacerbation frequency, and prognosis. HRCT often serves as the diagnostic gold standard, yet its visual interpretation remains subjective, with limited predictive value regarding treatment outcomes and disease progression [9].

Computer-aided diagnostic [CAD] systems aim to overcome these limitations by objectively quantifying lung abnormalities [10]. Previous research has demonstrated that certain HRCT features, such as Cystic bronchiectasis, correlate with worse lung function and HRCT scores [11,12,13]. However, studies have also shown a weak or absent correlation between HRCT scores and clinical parameters, such as lung function and quality of life assessments [12,13,14,15]. This discordance highlights the inadequacy of conventional HRCT in capturing the full complexity of bronchiectasis pathophysiology. As Artificial Intelligence [AI] continues to evolve, its application in medical imaging offers significant potential to augment HRCT interpretation. AI algorithms can enhance diagnostic precision, reveal latent disease patterns, objectively and quantitatively assess various spectrum of lung pathologies that can be identified in HRCT, and provide a more nuanced understanding of disease progression. By integrating AI into clinical practice, healthcare providers may improve diagnostic accuracy, optimize treatment decisions, and ultimately enhance patient outcomes [16]. This study aims to compare the capacity of AI to detect subtle and latent abnormalities in bronchiectasis, quantify radiologic abnormalities compared to traditional HRCT interpretations, and compare AI scoring with traditional bronchiectasis clinical scoring [BRICS].

## 2. Materials and Methods

### 2.1. Patients Selection

This was an observational retrospective study conducted at JSS Medical College and Hospital, Mysuru, Karnataka. This study was approved by the Institutional Ethics Committee [approval number: JSS/MC/PG/IEC-105/2022-23].

A total of 45 patients were enrolled in the Department of Respiratory Medicine, JSS Hospital, Mysuru, between June 2022 and June 2023, based on predefined inclusion criteria. Inclusion criteria were those with HRCT-confirmed bronchiectasis J47 [as per ICD 10]. As per exclusion criteria, those with active tuberculosis and active malignancy were excluded from the study. Comprehensive data regarding the patients’ general characteristics, comorbidities, smoking history, respiratory symptoms, and HRCT results were collected. The three experienced radiologists collated the Bronchiectasis Severity Score.

### 2.2. CT Scoring

The Bronchiectasis Radiologically Indexed CT Score [BRICS] is a severity assessment score for bronchiectasis that was created from a cohort of patients with idiopathic and post-infectious bronchiectasis by thoracic radiologists and chest physicians in Edinburgh, Scotland. As per the BRICS severity score assessment, subjects were classified into Mild [1], Moderate [2,3], and Severe [4,5] based on bronchial dilation and the number of bronchopulmonary segments affected by emphysema. The BRICS was chosen as it was simple, accurate, and predictive of illness severity as compared to other existing scores [17].

### 2.3. CT Scan Technique and Image Analysis

HRCT scans for the studies were acquired using the Philips Ingenuity 128-slice MDCT scanner. Only axial volumetric multidetector computed tomography [CT] scans taken in the supine position with a slice thickness of 3 mm, then reconstructed to 1 mm, were included for analysis.

QA [quantitative analysis] was performed in the set of images with a standard reconstruction algorithm by using Imbio LTA [based on the algorithm of CALIPER—Computer-aided Lung Informatics for Pathology Evaluation and Rating], a computational platform for the near-real-time characterization and quantification of lung parenchymal patterns on CT scans [18,19]. Lung parenchymal patterns include normal, hyperlucent, ground glass [GG], reticular, and honeycombing. Imbio LTA provided the Total Lung Volume, relative volumes, and absolute volumes of lung patterns, and their regional distribution within three different lung zones [upper, middle, lower] in each lung. The platform provided a fast output of the DICOM series, as well as a PDF file in which each lung pattern was colored differently, allowing the evaluation of disease extent, composition, and location at a glance. Each QA was performed automatically for up to 20 min [20]. Among the patterns detected by AI, hyperlucency is suggestive of air-trapping and alveolar destruction, while ground-glass opacity suggests active alveolitis and early interstitial inflammation. Both reticular and honeycombing are findings associated with fibrosis, while the former suggests interlobular interstitial thickening and the latter interstitial fibrosis with lung destruction and dilation of peripheral airspaces. The pulmonary vascular volume [PVV] refers to the total volume of all visible arteries and veins in the lungs, encompassing both blood and vessel walls. PVV is an important metric for assessing the severity of various lung disorders, such as pulmonary hypertension [PH] and other pulmonary vascular conditions [21,22].

### 2.4. Definitions

According to the WHO classification for BMI among Asians, people were classified as underweight, normal, overweight, and obese E66 [as per ICD 10]. The comorbidities such as type 2 diabetes mellitus E11 [as per ICD 10] and hypertension I10 [as per ICD 10] have been defined by WHO, while the definitions for congestive cardiac failure [CCF] and ischemic heart disease [IHD] have been given by the American College of Cardiology [ACC] and the American Heart Association [AHA]. We have also considered 5 types of bronchiectasis—Cystic, Saccular, varicose, Tubular or cylindrical, and traction bronchiectasis as per Reid classification [23]. The emphysema-dominant phenotype is an imaging-derived phenotype that is characterized by hyperlucency ≥ 20% [24]. Fibrosis’s predominant phenotype is often associated with fibrotic changes exceeding ≥20% in lung parenchyma [25]. Combined fibrosis and emphysema were subjects with a combination of both emphysema [≥20%] and fibrosis [≥20%] on chest CT scans [26].

### 2.5. Data Analysis

Data were entered into Microsoft Excel 2019 and analyzed using SPSS Version 26.0 [IBM Corp, released 2019, IBM SPSS Statistics for Windows, Version 26.0. Armonk, NY, USA: IBM Corp.]. The percentage was calculated for descriptive variables. The chi-square test was applied to analyze qualitative data. Mean and standard deviation were used to describe quantitative data. Pearson’s correlation was applied to analyze the correlation between BRICS and parenchymal changes detected by Artificial Intelligence. ANOVA was used to test the difference between parenchymal changes detected by Artificial Intelligence and the severity and type of Bronchiectasis. A *p*-value of <0.05 was considered to be statistically significant.

## 3. Results

Forty-five patients were included in this study. The majority were aged ≥60 years [48.9%] and the gender distribution was relatively balanced. More than one-third of the subjects were obese [19, 42.2%] with most participants [36, 80%] having comorbidities. Tractional bronchiectasis was the most common [35.6%], followed by nearly one-third [31.1%] with Moderate bronchiectasis, while 24.4% had Severe bronchiectasis and 8.9% had Mild bronchiectasis (Table 1).

Lung volume increases progressively from Mild [2.95 L] to Severe [3.85 L] bronchiectasis. The differences across severity levels are statistically significant [*p* = 0.038], suggesting a clear relationship between lung volume and disease severity and could suggest air trapping due to airway obstruction or emphysema. Hyperlucency, which reflects areas of lung tissue that appear abnormally transparent on imaging, increases significantly with severity, from 8.50% in Mild cases to 24.64% in Severe cases. The *p*-value of 0.025 indicates a statistically significant increase in lung hyperlucency as severity increases. Mild cases show the highest ground-glass opacity [22.50%], and the values for Moderate [14.93%] and Severe [16.27%] are lower. The *p*-value [0.477] indicates no statistically significant differences across severity levels for ground-glass opacity. Reticular opacity, which suggests fibrosis or scarring, increases with disease severity. Mild cases show 4.75%, while Severe cases reach 17.45%. Honeycombing, associated with advanced fibrosis, increases with severity, from 2.75% in Mild cases to 12.18% in Severe cases. PVV increases significantly [*p* = 0.04] as severity progresses, from 95 cm^3^ in Mild cases to 122.82 cm^3^ in Severe cases. Lung characteristics such as hyperlucency, reticular opacity, and PVV significantly increase with disease severity, indicating that these factors correlate with the progression of lung volume and increase progressively from Mild [2.95 L] to Severe [3.85 L] bronchiectasis. The differences across severity levels are statistically significant [*p* = 0.038], suggesting a clear relationship between lung volume and disease severity and could suggest air trapping due to airway obstruction or emphysema. Hyperlucency, which reflects areas of lung tissue that appear abnormally transparent on imaging, increases significantly with severity, from 8.50% in Mild cases to 24.64% in Severe cases. The *p*-value of 0.025 indicates a statistically significant increase in lung hyperlucency as severity increases. Mild cases show the highest ground-glass opacity [22.50%], and the values for Moderate [14.93%] and Severe [16.27%] are lower. The *p*-value [0.477] indicates no statistically significant differences across severity levels for ground-glass opacity. Reticular opacity, which suggests fibrosis or scarring, increases with disease severity. Mild cases show 4.75%, while Severe cases reach 17.45%. Honeycombing, associated with advanced fibrosis, increases with severity, from 2.75% in Mild cases to 12.18% in Severe cases. PVV increases significantly [*p* = 0.04] as severity progresses, from 95 cm^3^ in Mild cases to 122.82 cm^3^ in Severe cases. Lung characteristics such as hyperlucency, reticular opacity, and PVV significantly increase with disease severity, indicating that these factors correlate with the progression of lung pathology. Lung volume and honeycombing also increase, but the differences in honeycombing are not statistically significant. Ground-glass opacity does not show a clear trend or significant change with severity (Table 2).

Total Lung Volume [L] is highest in Tubular bronchiectasis [3.875 L] and lowest in Saccular bronchiectasis [2.963 L], suggesting greater air trapping or hyperinflation in Tubular types. Total Lung Hyperlucency follows a similar trend, being highest in the Tubular [23.5%] and lowest in the Saccular [6.62%], with elevated hyperlucency also observed in Cystic types. Ground-glass opacity [GGO] is most prominent in Saccular bronchiectasis [26.87%] and least in Tubular [1.5%], potentially reflecting active inflammation or early fibrosis in Saccular types. Reticular opacity is highest in Cystic bronchiectasis [13.27%] and lowest in Tubular [3.5%], while honeycombing—a marker of advanced fibrosis—is most pronounced in Tubular [13.25%] and least in Saccular [2.87%]. Pulmonary Vessel Volume [PVV], reflecting blood vessel volume, is elevated in Cystic bronchiectasis, suggesting increased vascular involvement. Overall, Cystic and Tubular bronchiectasis are associated with higher lung volumes, hyperlucency, and PVV, which is indicative of more advanced lung changes, while Saccular and tractional types show relatively lower values. These findings reveal emerging patterns of lung alterations specific to bronchiectasis types, highlighting a trend toward more severe structural changes in Cystic and Tubular bronchiectasis (Table 3).

AI appears to detect abnormalities at a higher rate than traditional radiology in all categories. The most significant difference is in the detection of lung hyperlucency, where AI detects 88.9% compared to radiology’s 24.4%, while for ground-glass opacity, the detection rate of AI is 82.2% compared to conventional radiology [26.7%] (Table 4).

**Figure 1:**
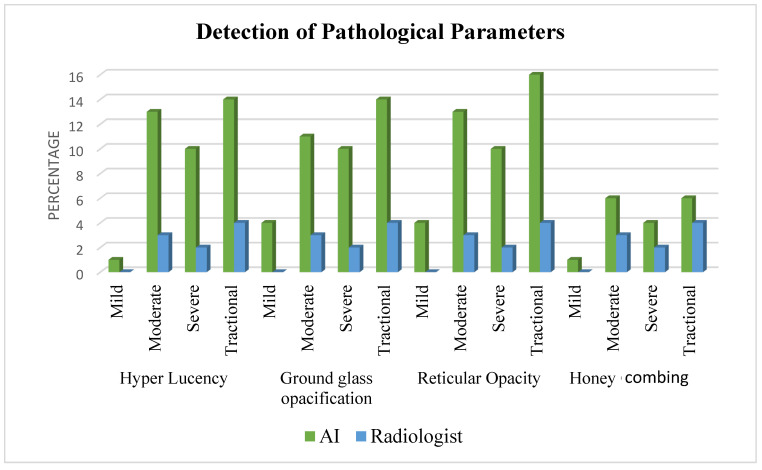
Detection of parenchymal pathologies by AI and radiologist.

The AI consistently detected more cases of parenchymal pathologies compared to the radiologist, especially in more Severe and tractional cases (Figure 1).

**Figure 2:**
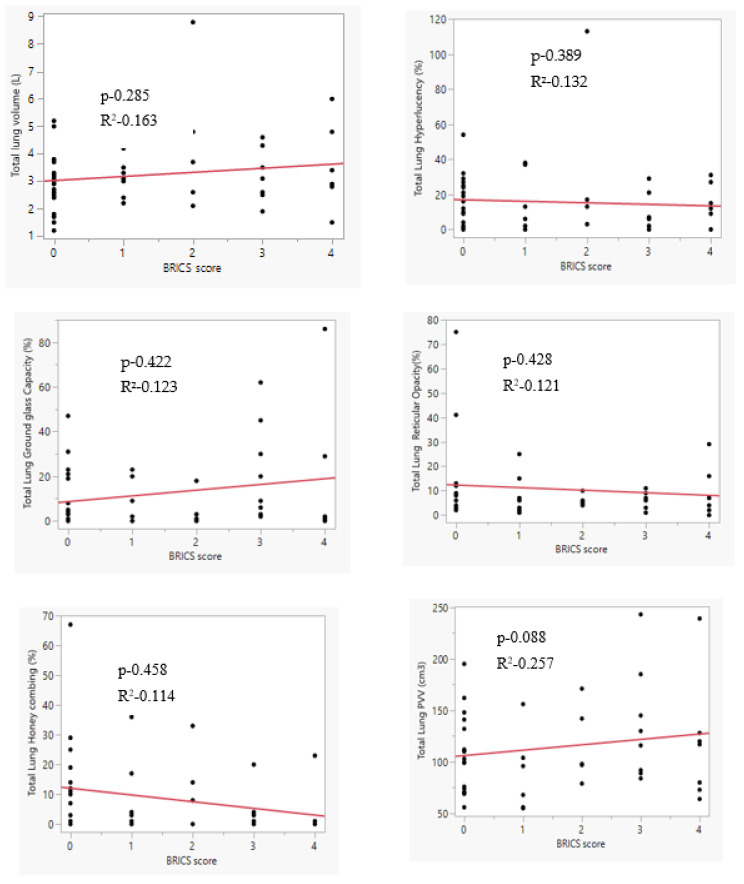
Correlation between various parenchymal pathologies detected by Artificial Intelligence and BRICS.

The correlation between AI-detected pathologies and BRICSs is weak (Figure 2).

**Figure 3:**
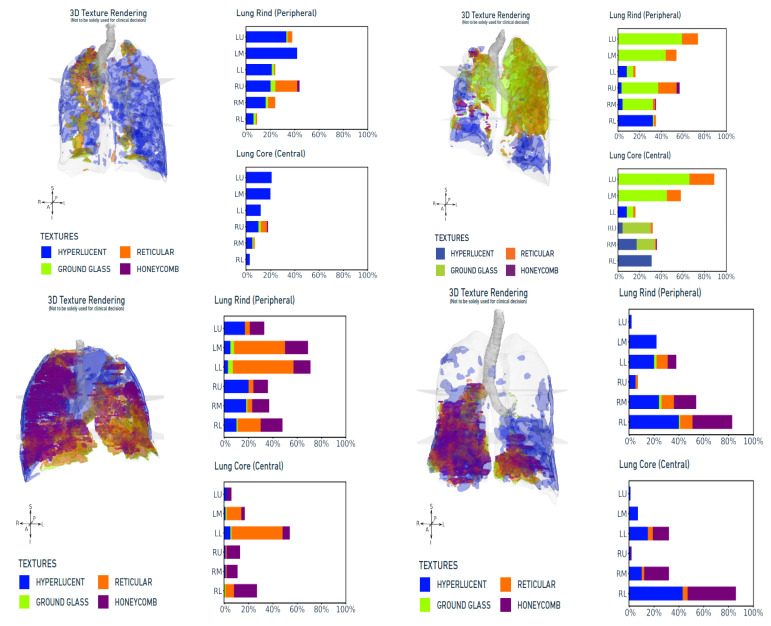
Lung texture analysis report: The Dicom representative image of each category of pattern. These were chosen based on the predominance of lung parenchymal patterns including hyperlucent, ground glass [GG], reticular, and honeycombing. Imbio LTA provided their regional distribution within three different lung zones [upper, middle, lower] in each lung. The platform provided an image in which each lung pattern was colored differently, allowing the evaluation of disease extent, composition, and location at a glance. Specific images were chosen because of the predominance of one of the patterns in each of them; at the same time, they had other patterns, also showing that all pathologies could coexist in a single case of bronchiectasis, yet have a predominance of a single pattern that may have a bearing on their clinical symptoms.

In our study, we identified 16 [35.5%] patients with hyperlucency exceeding 20% [smokers—6 patients, non-smokers—10 patients], and this can be classified as an emphysema-predominant phenotype. Additionally, 7 [15%] patients presented with combined reticular shadows and honeycombing above 20%, and can be classified as a fibrosis-predominant phenotype, and 2 [4.4%] of the subjects satisfied the criteria for combined emphysema and fibrosis [24,25,26,27,28]. About 33% of patients had significant ground-glass opacities and follow-up studies are needed to understand the natural history of these patients (Figure 3).

## 4. Discussion

This study provides an important analysis of the use of Artificial Intelligence [AI] in interpreting HRCT scans of bronchiectasis. While numerous studies have examined the relationships between functional status, spirometry, quality of life, and HRCT findings in bronchiectasis, to the best of our knowledge, none have explored the potential of AI to enhance the diagnostic and prognostic capabilities of bronchiectasis. Our findings demonstrate that AI can significantly augment the detection of radiological phenotypes in bronchiectasis patients and provide quantitative estimation compared to traditional radiology alone, making this study novel and impactful. In line with findings from the EMBARC India Registry, a significant portion of bronchiectasis cases in our cohort were post-infectious, primarily following tuberculosis. AI-driven lung texture analysis identified specific radiological patterns—hyperlucency, ground-glass opacity [GGO], reticular shadows, and honeycombing—at higher rates than those observed by radiologists. Additionally, this AI-based analysis provided quantitative estimates of the proportion of lungs affected by each of these patterns. These radiological patterns offer valuable insights into the underlying pathophysiology of bronchiectasis and its associated pulmonary abnormalities.

Hyperlucency in the lungs in bronchiectasis can arise from a variety of structural and pathological changes, including emphysema, and vascular anomalies [29]. AI detected hyperlucency in 88.9% of cases, with the highest incidence in Tubular bronchiectasis [23.5%]. This rate was significantly higher than the 24.4% detection rate by radiologists, underscoring AI’s potential to capture subtle emphysematous alterations. Supporting studies by Loubeyre et al. suggest that these emphysematous changes may result from bronchiolar inflammation, which disproportionately affects peripheral airways and may increase susceptibility to emphysema in advanced bronchiectasis [30]. Increased neutrophil elastase in the sputum or BAL is associated with increased hyperlucency [31,32,33]. The severity of the emphysema appears to be the most important determinant of the chronic deterioration of airflow [34,35,36].

Ground-glass opacity [GGO], often indicative of decreased air content in the lung parenchyma without complete alveolar obstruction, is associated with reversible conditions such as minimal alveolar septal thickening or partial fluid accumulation. In our study, AI detected GGO in 82.2% of cases, with the highest prevalence in Saccular bronchiectasis [26.85%]. The presence of GGO may reflect an ongoing inflammatory response or infectious process, highlighting AI’s capacity to detect early, potentially reversible changes in bronchiectasis pathology [37].

Reticular opacities—characterized by a network of linear densities often linked with interstitial lung diseases—were identified in 55.6% of cases by AI, in contrast to only 6.7% detected by three radiologists. This substantial discrepancy demonstrates AI’s heightened sensitivity in identifying interstitial changes, which could have significant implications for diagnosing fibrotic conditions in bronchiectasis. While reticular shadows appeared across all bronchiectasis types, slightly higher values were noted in cystic and tractional forms [38].

Honeycombing, a hallmark of end-stage fibrotic lung disease, presents as clustered cystic airspaces. AI detected honeycombing in 60% of cases compared to 13.3% by radiologists. This notable difference suggests that AI could play a critical role in identifying advanced fibrotic changes that may be challenging to detect visually, even by experienced radiologists [38]. The AI-detected patterns align with the histopathological features of bronchiectasis, such as alveolar collapse and dilation of alveolar ducts, further validating AI’s utility in identifying advanced disease stages [39].

While HRCT can assess the type and severity of bronchiectasis, AI algorithms offer the capacity to analyze vast datasets, uncovering patterns and associations that may elude human observers. By integrating data from multiple sources, including imaging and clinical records, AI provides a more comprehensive view of bronchiectasis, aiding in the identification of disease phenotypes. In our study, we identified the following three main radiological phenotypes: bronchiectasis with emphysema and bronchiectasis with interstitial involvement [evidenced by ground-glass opacity, reticular patterns, and honeycombing], and the third with both emphysema and fibrosis. Recognizing bronchiectasis as a heterogeneous disease and not just airway disease helps explain the broad spectrum of clinical symptoms and variability in severity, which is potentially linked to these distinct radiological phenotypes, and may inform future clinical studies to address these pathologies in bronchiectasis [40].

Advancements in AI-based algorithms for monitoring and assessing bronchiectasis align with broader progress across medical fields. For instance, a study by Díaz et al. utilizing AI in chest CT imaging from the COPD Gene cohort examined bronchiectasis in COPD, particularly focusing on the airway-to-artery ratio [AAR] [41]. This study found that a higher AAR [greater than 1] correlated with increased exacerbation frequency in COPD patients, suggesting AI’s potential in early bronchiectasis detection and exacerbation risk prediction. However, our study uniquely employs the IMBIO software’s lung texture analysis tool to detect subtle patterns that may be overlooked by radiologists, highlighting the novelty of our approach [42].

Our study’s strengths include a bronchiectasis-specific cohort, enabling an in-depth analysis of the links between radiographic abnormalities and clinically meaningful outcomes. Nonetheless, there are limitations to consider. Radiographic scores represent a single time point and do not capture the dynamic nature of bronchiectasis, as radiographic abnormalities can vary over time. Additionally, the widespread application of these scoring methods is challenging due to the need for specialized expensive software. AI technologies are still in an early stage of development and are yet to be fully integrated into routine clinical practice [41,43].

The integration of AI into clinical workflows also requires thoughtful planning to ensure seamless alignment with existing radiological practices. Effective implementation will necessitate training for radiologists and healthcare professionals to use AI tools in a way that complements, rather than disrupts, the diagnostic process [44]. Addressing these challenges will require a collaborative effort among researchers, radiologists, and clinicians to facilitate the adoption of AI in a comprehensive evaluation of bronchiectasis. With further research, AI holds the potential to transform the early detection and prognosis of bronchiectasis, ultimately enhancing patient outcomes [45]. Additionally, further clinical research is needed to identify the ideal therapeutic modalities for the two main radiologic phenotypes identified in this study, the emphysema-predominant bronchiectasis [role of neutrophil elastase inhibitors], and the interstitial fibrosis-predominant bronchiectasis [role of anti-fibrotics]. The natural course of ground-glass opacity is quite unclear and further longitudinal studies would be needed to conclude the phenotype into which it can be eventually categorized.

In summary, our findings underscore AI’s potential to enhance the diagnostic precision of HRCT in bronchiectasis by delivering more consistent, detailed quantitative measurements and objective evaluations of disease-specific radiological characteristics. AI’s superior performance in detecting hyperlucency, GGO, reticular shadows, and honeycombing suggests that it could serve as a valuable adjunct to traditional radiology, potentially guiding more tailored therapeutic strategies. Future research should further explore AI’s role in assessing disease progression and response to treatment, which could ultimately enhance patient outcomes and quality of life.

## Figures and Tables

**Table 1:** Socio-demographic profile of the study participants [*n* = 45].

Socio-Demographic Profile		Number [%]
Age [in years]	≤40	4 [8.9%]
	40–60	19 [42.2%]
	≥60	22 [48.9%]
Gender	Male	23 [51.1%]
	Female	22 [48.9%]
BMI [kg/m^2^] [As per WHO]	Underweight	6 [13.3%]
	Normal	11 [24.4%]
	Overweight	9 [20.0%]
	Obese	19 [42.2%]
Comorbidities	Hypertension [ICD I10]	11 [24.4%]
	Type 2 DM [ICD E11.9]	14 [31.1%]
	Hypertension and Type 2 DM	5 [11.1%]
	Congestive Cardiac Failure [ICD I50]	1 [2.2%]
	Hypothyroid [ICD E03.9]	2 [4.4%]
	Ischemic Heart Disease [ICD I25]	3 [6.6%]
	No Comorbidities	9 [20%]
Severity of Bronchiectasis based on Bronchiectasis Radiologically Indexed CT Score [BRICS]	Mild	4 [8.9%]
	Moderate	14 [31.1%]
	Severe	11 [24.4%]
	Tractional	16 [35.6%]
Smoking Status	Smoker	14 [31.1%]
	Non-smoker	31 [68.9%]
Symptoms	Cough	29 [64.4%]
	Fever	16 [35.5%]
	Shortness of Breath	22 [48.8%]
	Hemoptysis	2 [4.44%]

**Table 2:** Relationship between various parenchymal pathologies detected by Artificial Intelligence and severity of Bronchiectasis.

Severity		Mean ± Std. Deviation	Std. Error	95% Confidence Interval for Mean	*p*-Value
Total Lung Volume [L]	Mild	2.950 ± 1.0214	0.5107	1.325–4.575	0.038
	Moderate	3.200 ± 0.8806	0.2353	2.692–3.708	
	Severe	3.845 ± 2.0863	0.6290	2.444–5.247	
	Tractional	2.969 ± 1.0562	0.2641	2.406–3.532	
Total Lung Hyperlucency [%]	Mild	8.50 ± 15.695	7.848	−16.47–33.47	0.025
	Moderate	9.79 ± 11.696	3.126	3.03–16.54	
	Severe	24.64 ± 31.646	9.542	3.38–45.90	
	Tractional	16.19 ± 13.949	3.487	8.75–23.62	
Total Lung Ground-glass Opacity [%]	Mild	22.50 ± 19.261	9.631	−8.15–53.15	0.477
	Moderate	14.93 ± 18.805	5.026	4.07–25.79	
	Severe	16.27 ± 14.158	7.887	10.692–24.85	
	Tractional	7.63 ± 9.966	2.491	2.31–12.94	
Total Lung Reticular Opacity [%]	Mild	4.75 ± 2.754	1.377	0.37–9.13	0.05
	Moderate	7.21 ± 6.302	1.684	3.58–10.85	
	Severe	17.45 ± 15.885	5.393	5.585–25.47	
	Tractional	17.38 ± 15.850	4.712	4.04–23.42	
Total Lung Honeycombing [%]	Mild	2.75 ± 5.500	2.750	−6.00–11.50	0.328
	Moderate	9.86 ± 4.646	2.578	5.784–10.43	
	Severe	12.18 ± 9.040	3.630	8.23–17.27	
	Tractional	12.88 ± 17.366	4.342	3.62–22.13	
Total Lung PVV [cm^3^]	Mild	95.00 ± 44.803	22.402	23.71–166.29	0.04
	Moderate	117.50 ± 49.640	13.267	88.84–146.16	
	Severe	122.82 ± 53.961	16.270	86.57–159.07	
	Tractional	110.00 ± 37.932	9.483	89.79–130.21	

**Table 3:** Relationship between various parenchymal pathologies detected by Artificial Intelligence and type of bronchiectasis.

Type of Bronchiectasis			Mean ± Std. Deviation	Std. Error	95% Confidence Interval for Mean	*p*-Value
Total Lung Volume [L]	Saccular	8	2.963 ± 1.0501	0.3713	2.085–3.840	0.641
	Cystic	11	3.555 ± 1.8976	0.5721	2.280–4.829	
	Tubular	4	3.875 ± 0.8098	0.4049	2.586–5.164	
	Varicoid	6	3.433 ± 1.5423	0.6296	1.815–5.052	
	Tractional	16	2.969 ± 1.0562	0.2641	2.406–3.532	
Total Lung Hyperlucency [%]	Saccular	8	6.625 ± 9.6501	3.4118	−1.443–14.693	0.446
	Cystic	11	21.455 ± 32.2222	9.7154	−0.193–43.102	
	Tubular	4	23.500 ± 17.2916	8.6458	−4.015–51.015	
	Varicoid	6	9.833 ± 11.8561	4.8402	−2.609–22.276	
	Tractional	16	16.188 ± 13.9486	3.4871	8.755–23.620	
Total Lung Ground-glass Opacity [%]	Saccular	8	26.875 ± 31.1698	11.0202	0.816–52.934	0.106
	Cystic	11	13.727 ± 15.4861	4.6692	3.324–24.131	
	Tubular	4	1.500 ± 3.0000	1.5000	−3.274–6.274	
	Varicoid	6	12.167 ± 18.2254	7.4405	−6.960–31.293	
	Tractional	16	7.625 ± 9.9658	2.4914	2.315–12.935	
Total Lung Reticular Opacity [%]	Saccular	8	8.625 ± 8.8307	3.1221	1.242–16.008	0.671
	Cystic	11	13.273 ± 17.2921	5.2138	1.656–24.890	
	Tubular	4	3.500 ± 2.6458	1.3229	−0.710–7.710	
	Varicoid	6	6.500 ± 5.5767	2.2767	0.648–12.352	
	Tractional	16	13.375 ± 18.8498	4.7125	3.331–23.419	
Total Lung Honeycombing [%]	Saccular	8	2.875 ± 8.1317	2.8750	−3.923–9.673	0.398
	Cystic	11	6.091 ± 6.4568	1.9468	1.753–10.429	
	Tubular	4	13.250 ± 15.1079	7.5540	−10.790–37.290	
	Varicoid	6	6.167 ± 14.6208	5.9689	−9.177–21.510	
	Tractional	16	12.875 ± 17.3662	4.3415	3.621–22.129	
Total Lung PVV [cm^3^]	Saccular	8	104.250 ± 35.1110	12.4136	74.896–133.604	0.561
	Cystic	11	134.545 ± 59.5472	17.9542	94.541–174.550	
	Tubular	4	101.500 ± 58.2666	29.1333	8.785–194.215	
	Varicoid	6	109.333 ± 42.9216	17.5227	64.290–154.377	
	Tractional	16	110.000 ± 37.9315	9.4829	89.788–130.212	

**Table 4:** Pathological parameters detected by radiology and AI.

	Radiology		AI	
	Detected	Not Detected	Detected	Not Detected
Total Lung Hyperlucency	11 [24.4%]	34 [75.6%]	40 [88.9%]	5 [11.1%]
Total Ground Glass	12 [26.7%]	33 [73.3%]	37 [82.2%]	8 [17.8%]
Total Reticular Opacity	3 [6.7%]	43 [93.3%]	25 [55.6%]	20 [44.4%]
Total Honeycombing	6 [13.3%]	39 [86.7%]	27 [60%]	18 [40%]

## Data Availability

The original contributions presented in this study are included in the article. Further inquiries can be directed to the corresponding author.
